# Supplementary material for: Dopey-dependent regulation of extracellular vesicles maintains neuronal morphology
Source: bioRxiv. 2024 May 8:2024.05.07.591898. Preprint. [Version 1] doi: 10.1101/2024.05.07.591898 (PMC11100700; doi:10.1101/2024.05.07.591898)
Supplement: 2 [file NIHPP2024.05.07.591898V1-supplement-1.pdf]

bioRxiv preprint doi: https://doi.org/10.1101/2024.05.07.591898; this version posted May 8, 2024. The copyright holder for this preprint (which was not certified by peer review) is the author/funder, who has granted bioRxiv a license to display the preprint in perpetuity. It is made available under aCC-BY 4.0 International license.

Supplemental Figures

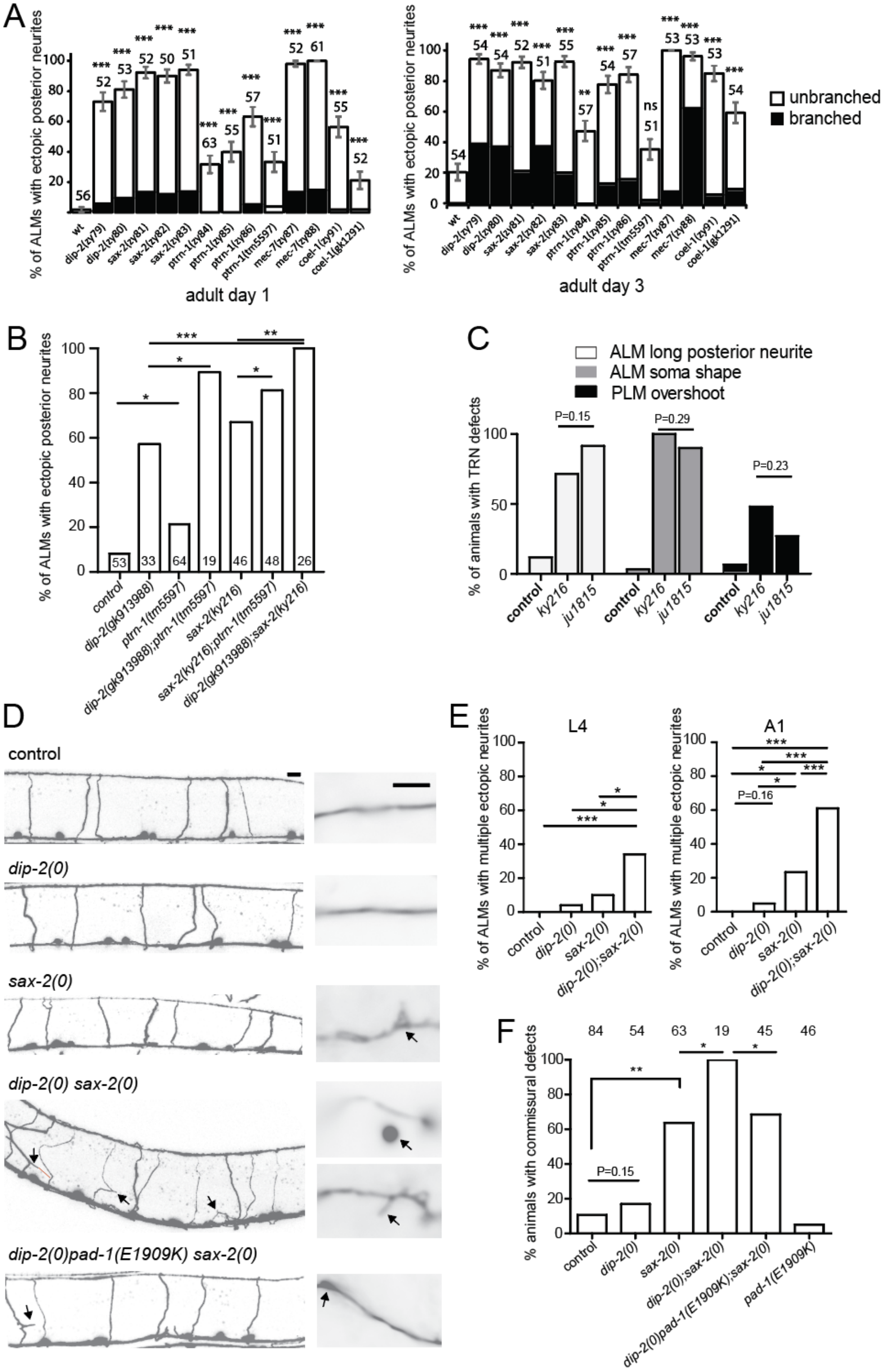

bioRxiv preprint doi: https://doi.org/10.1101/2024.05.07.591898; this version posted May 8, 2024. The copyright holder for this preprint (which was not certified by peer review) is the author/funder, who has granted bioRxiv a license to display the preprint in perpetuity. It is made available under aCC-BY 4.0 International license.

Supplemental Figure 1. *dip-2* displays specific synergistic interaction with *sax-2* in maintenance of neuron morphology

(A) Quantitation of ALM ectopic posterior neurites in mutants at 1-day (*left*) and 3-day (*right*) of adulthood. Mutants with *dip-2*-like phenotypes were isolated in a forward mutagenesis screen. Ectopic neurites were classified as branched or unbranched. Statistics for A: bars show mean and error bars indicate SEs of proportion for the indicated number of animals. Significance is compared to controls (*zdIs5*) using a one-way ANOVA with Tukey test for multiple comparisons. (B) Genetic interactions between *dip-2* and null mutations in genes involved in TRN neuronal morphology maintenance. *dip-2* and *sax-2* displayed the strongest synergistic interaction. ALM ectopic neurites were scored in 1-day old adults, 1 ALM per animal, transgenic marker *zdIs5*. N, number of ALMs scored. (C) Comparison of between *sax-2(ju1815)* deletion and *sax-2(ky216)* in TRN defects. Both mutations caused similar levels of morphological defects. N = 40-50 per genotype. (D) Confocal images (*left*) of D-type GABAergic motor neurons labelled with P*unc-25*-GFP(*juIs76*). *dip-2(0)* single mutants did not exhibit significant defects in D-type neuron morphology; in *sax-2(0)* single mutants, D-type neurons occasionally exhibited kinks, blebs, or round protrusions in their lateral commissures. In *dip-2(0) sax-2(0)* double mutants, D-type motor neurons displayed fully penetrant defects in commissural morphology as well as ectopic neurite sprouting in the ventral nerve cord (arrows). Scale = 10 μm. Details of commissure morphology are shown in enlarged insets (right); scale = 1 μm. Black arrows indicate defects including ectopic neurites, blebs, and vesicular structures. Confocal z-stacks were projected with maximum intensity. (E) Quantitation of ALM neurites with multiple branches in animals of genotype indicated. *dip-2(0) sax-2(0)* double mutants displayed synergistic increases in multiply branched ALM neurites in L4 and day 1 adult (A1). (F) Quantitation of commissural defects as % of animals with at least one defective commissure, scored in L4 stage. Statistics for panels B,C,E, and F: Fisher's exact test. *** (P<0.001), ** (P<0.01),* (P<0.05).

bioRxiv preprint doi: https://doi.org/10.1101/2024.05.07.591898; this version posted May 8, 2024. The copyright holder for this preprint (which was not certified by peer review) is the author/funder, who has granted bioRxiv a license to display the preprint in perpetuity. It is made available under aCC-BY 4.0 International license.

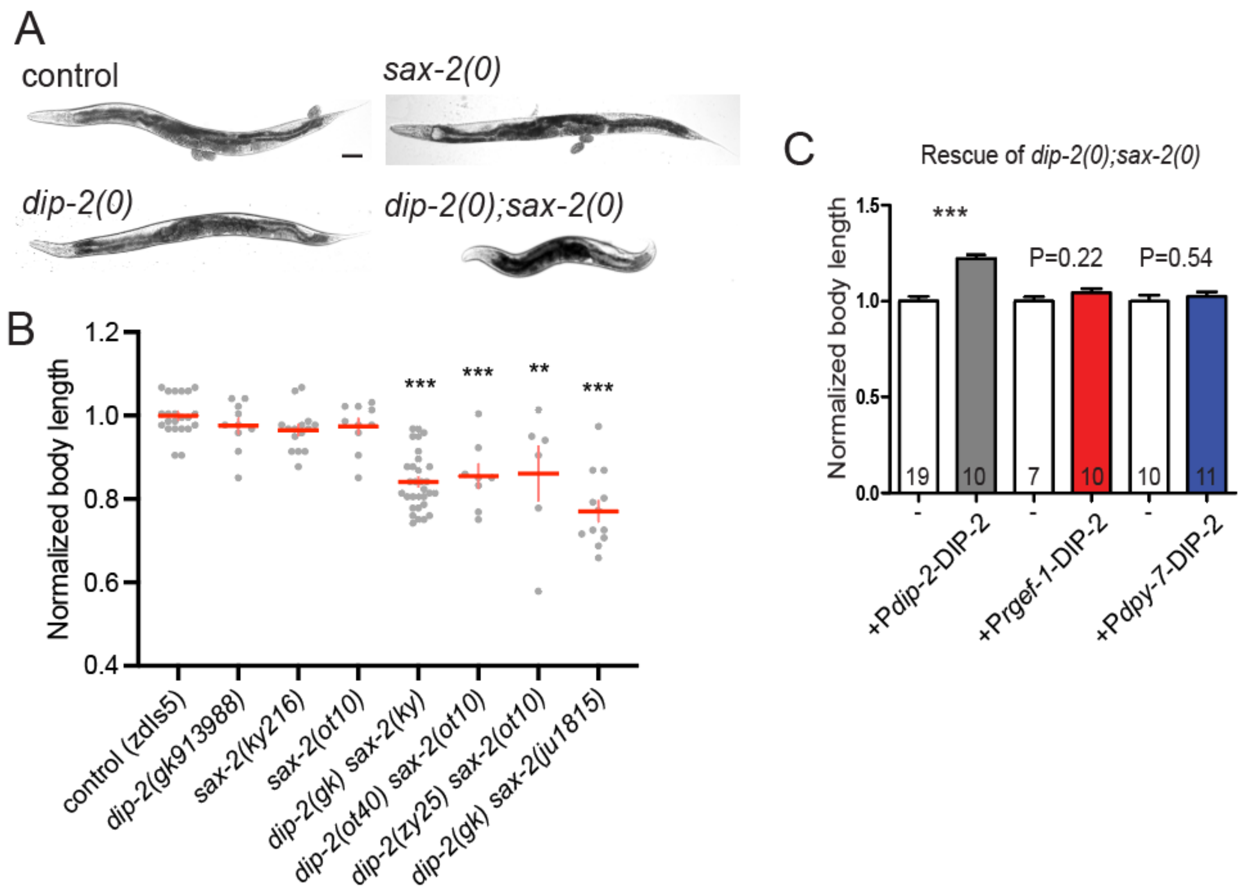

Supplemental Figure 2. *dip-2 sax-2* double mutants display synergistic effects on body length

(A) Bright field images of 1-day old adults of genotype indicated. *dip-2(0)* or *sax-2(0)* single mutants have normal morphology and body length, whereas *dip-2(0) sax-2(0)* double mutants are shorter in body length. Scale = 100 µm. (B) Quantitation of normalized body length in 1-day old adult animals; *zdIs5* background except for *dip-2(gk913988) sax-2(ju1815)*. All *dip-2 sax-2* double mutant combinations were significantly different from controls and were not significantly different from each other. N = 6-31 per genotype. Statistics: one-way ANOVA with Tukey post test. *** (P<0.001). (C) Quantitation of body length in 1-day old adult *dip-2(0) sax-2(0)* double mutants with and without transgenes, normalized to *dip-2(0) sax-2(0)* control. Body length defects of *dip-2(0) sax-2(0)* double mutants were rescued by overexpression of DIP-2 using its own promoter but not using the pan-neuronal *rgef-1* or the epidermal *dpy-7* promoters. Statistics: t test. *** (P<0.001).

bioRxiv preprint doi: https://doi.org/10.1101/2024.05.07.591898; this version posted May 8, 2024. The copyright holder for this preprint (which was not certified by peer review) is the author/funder, who has granted bioRxiv a license to display the preprint in perpetuity. It is made available under aCC-BY 4.0 International license.

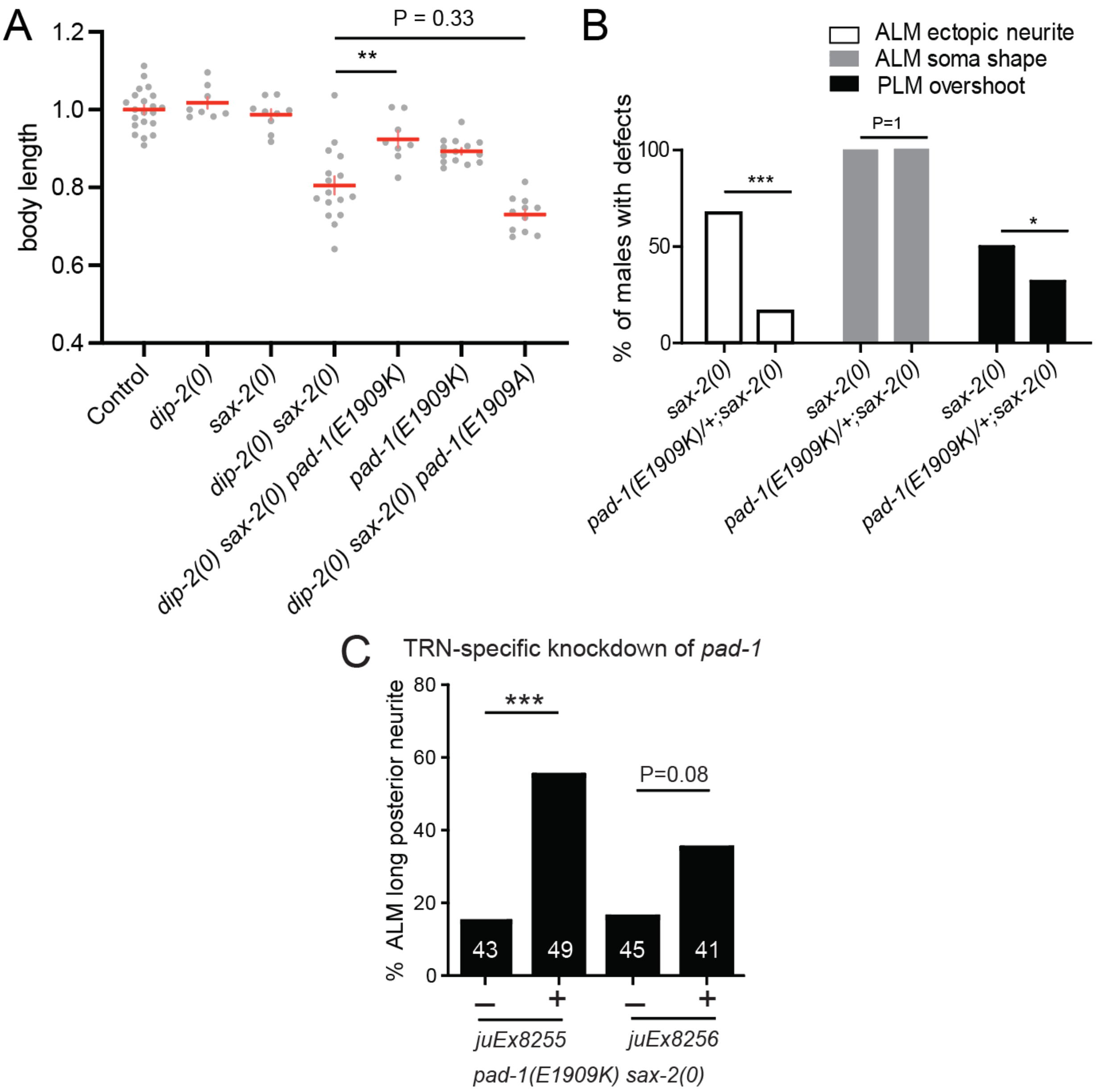

Supplemental Figure 3. Suppression of *dip-2 sax-2* double mutant and *sax-2* single mutant phenotypes by *pad-1(E1909K)*

(A) Quantitation of body length in 1-day old adults. *pad-1(E1909K)* but not *pad-1(E1909A)* suppressed the short body length of *dip-2(0) sax-2(0)* double mutants. N = 5-15 per genotype; normalized to wild type controls. Statistics: t-test. (B) Quantitation of morphological defects of touch neurons in *sax-2(0)* mutant males with heterozygous *pad-1(ju1806)*. *pad-1(ju1806)* acted as a semi-dominant suppressor of *sax-2* ALM ectopic neurite and PLM overshooting defects. N= 45-59. Statistics: Fisher exact test. *** (P<0.001),* (P<0.05). (C) TRN-specific dsRNA-mediated knockdown (KD) of *pad-1*

bioRxiv preprint doi: https://doi.org/10.1101/2024.05.07.591898; this version posted May 8, 2024. The copyright holder for this preprint (which was not certified by peer review) is the author/funder, who has granted bioRxiv a license to display the preprint in perpetuity. It is made available under aCC-BY 4.0 International license.

reverses the suppression of ectopic neurite outgrowth. *pad-1* KD in *pad-1(ju1806) sax-2(0)* increased % of ALMs with ectopic neurite outgrowth. Statistics: Fisher exact test. ***(P < 0.001), ** (P<0.01),* (P<0.05).

bioRxiv preprint doi: https://doi.org/10.1101/2024.05.07.591898; this version posted May 8, 2024. The copyright holder for this preprint (which was not certified by peer review) is the author/funder, who has granted bioRxiv a license to display the preprint in perpetuity. It is made available under aCC-BY 4.0 International license.

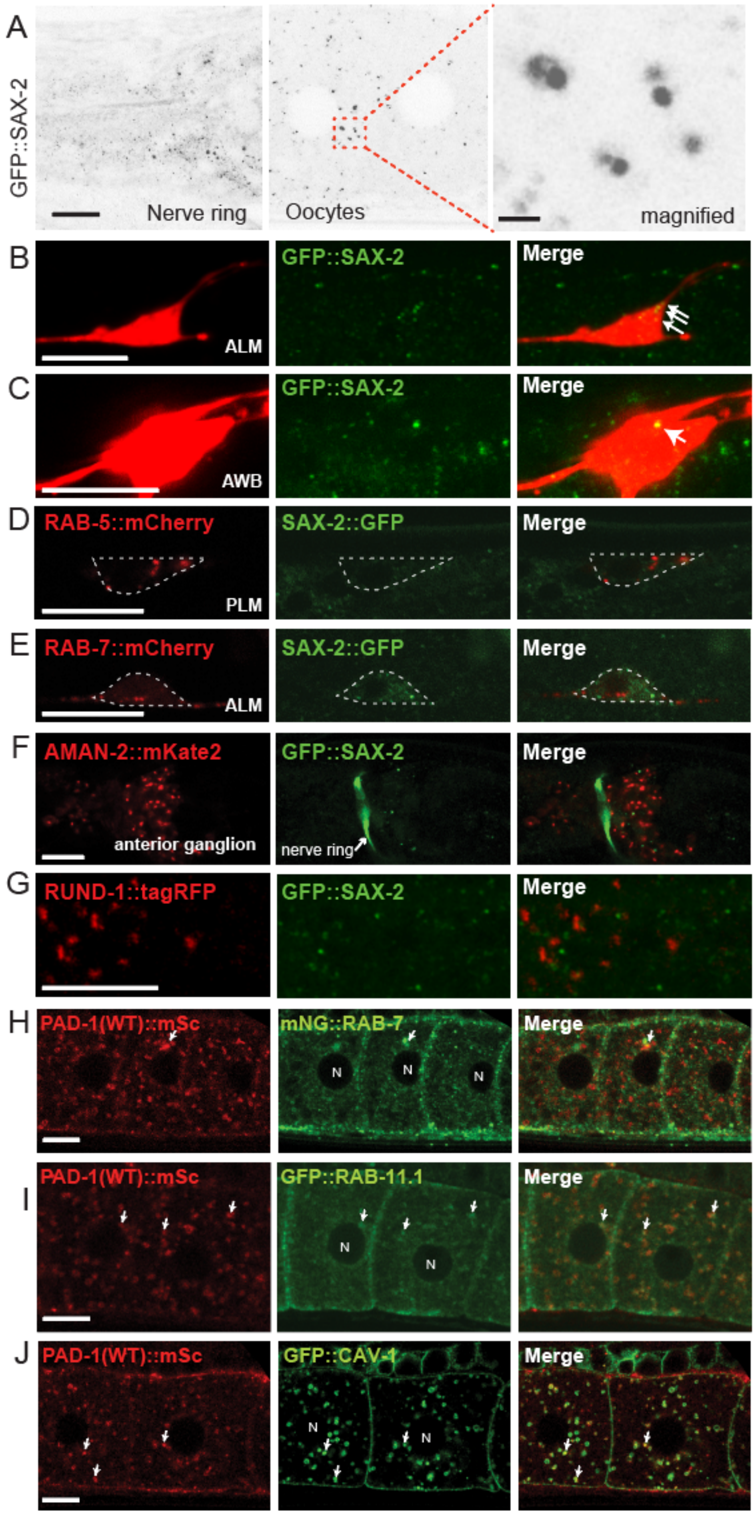

Supplemental Figure 4. Localization of SAX-2::GFP to subcellular puncta in neurons and oocytes and lack of co-localization with endosome or Golgi markers

bioRxiv preprint doi: https://doi.org/10.1101/2024.05.07.591898; this version posted May 8, 2024. The copyright holder for this preprint (which was not certified by peer review) is the author/funder, who has granted bioRxiv a license to display the preprint in perpetuity. It is made available under aCC-BY 4.0 International license.

(A) Confocal images of GFP::SAX-2 (*syb3389*) knock-in expression in nerve ring and oocytes; maximum intensity projections of 3 focal planes, scale = 10 µm. Inset shows SAX-2 puncta magnified; scale = 1 µm. (B) GFP::SAX-2 forms small puncta (arrows) in peripheral soma of ALM neuron labeled with mCherry (*juEx5179*). (C) GFP::SAX-2 formed a single large punctum in soma of sensory neuron AWB (arrow; red marker = *oyIs65*). Maximum intensity projections of 15 focal planes, scale = 10 µm. (D-E) Confocal images of TRNs and nerve ring region co-expressing GFP::SAX-2 or SAX-2::GFP KI with red fluorescent markers: RAB-5::mCherry for early endosomes, RAB-7::mCherry for late endosomes, AMAN-2::mKate2 for medial Golgi, and RUND-1::tagRFP for *trans* Golgi. In images displaying the nerve ring labelled with GFP::SAX-2 and AMAN-2::mKate2 (*juEx8137*), AIY is labeled green due to P*ttx-3*-GFP co-injection marker. (H-J) Confocal images of oocytes co-expressing PAD-1::mSc and green fluorescent labels for intracellular organelles. PAD-1::mSc (white arrows) was partly co-localized with mNG::RAB-7 KI (*utx12*) and RAB-11.1 marking late and recycling endosomes, respectively. Vesicular PAD-1::mSc also partially co-localized with *pie-1*-driven GFP::CAV-1 (*pwIs28*), a component of caveolae and of cortical granules in oocytes [79]. White arrows indicate punctate colocalization; N indicates nucleus. For panels B-J, scale = 10 µm.

bioRxiv preprint doi: https://doi.org/10.1101/2024.05.07.591898; this version posted May 8, 2024. The copyright holder for this preprint (which was not certified by peer review) is the author/funder, who has granted bioRxiv a license to display the preprint in perpetuity. It is made available under aCC-BY 4.0 International license.

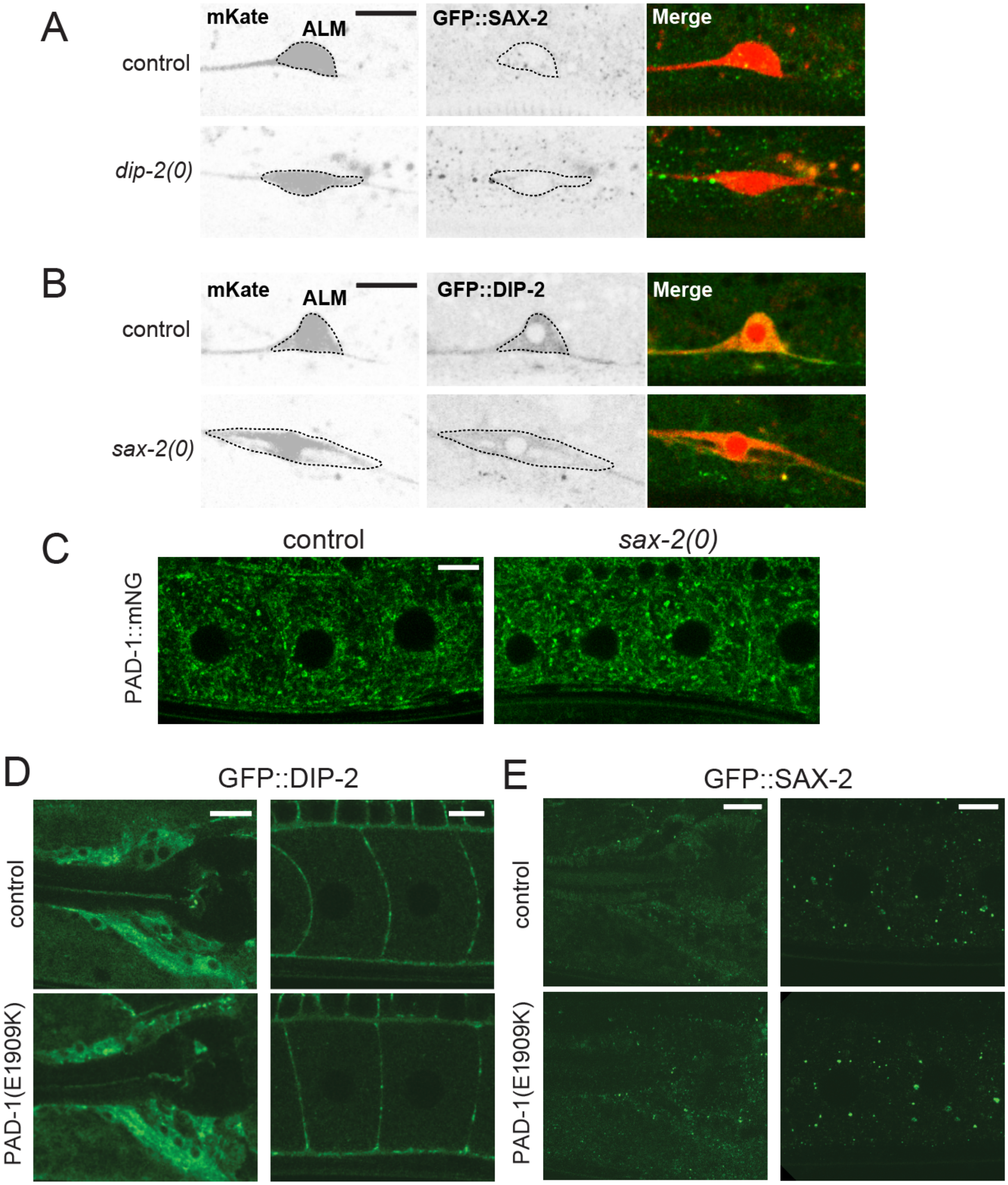

Supplemental Figure 5. Localization of DIP-2, SAX-2, and PAD-1 in neurons and oocytes

(A) Confocal images of ALMs expressing GFP::SAX-2 KI. SAX-2 localization in ALM was not altered in *dip-2(0)* mutants. (B) GFP::DIP-2 KI localization in ALM was not

bioRxiv preprint doi: https://doi.org/10.1101/2024.05.07.591898; this version posted May 8, 2024. The copyright holder for this preprint (which was not certified by peer review) is the author/funder, who has granted bioRxiv a license to display the preprint in perpetuity. It is made available under aCC-BY 4.0 International license.

altered in *sax-2(0)* mutants. (C) Confocal images of oocytes expressing PAD-1::mNG KI. In *sax-2(0)* mutants, PAD-1::mNG KI expression was not significantly different from wild type. (D) The expression pattern and level of GFP::DIP-2 in both neurons and oocytes were not altered in *pad-1(E1909K)* mutants. (E) PAD-1(E1909K) did not affect GFP::SAX-2 in both neurons and oocytes. For all panels, scale = 10 μm.

bioRxiv preprint doi: https://doi.org/10.1101/2024.05.07.591898; this version posted May 8, 2024. The copyright holder for this preprint (which was not certified by peer review) is the author/funder, who has granted bioRxiv a license to display the preprint in perpetuity. It is made available under aCC-BY 4.0 International license.

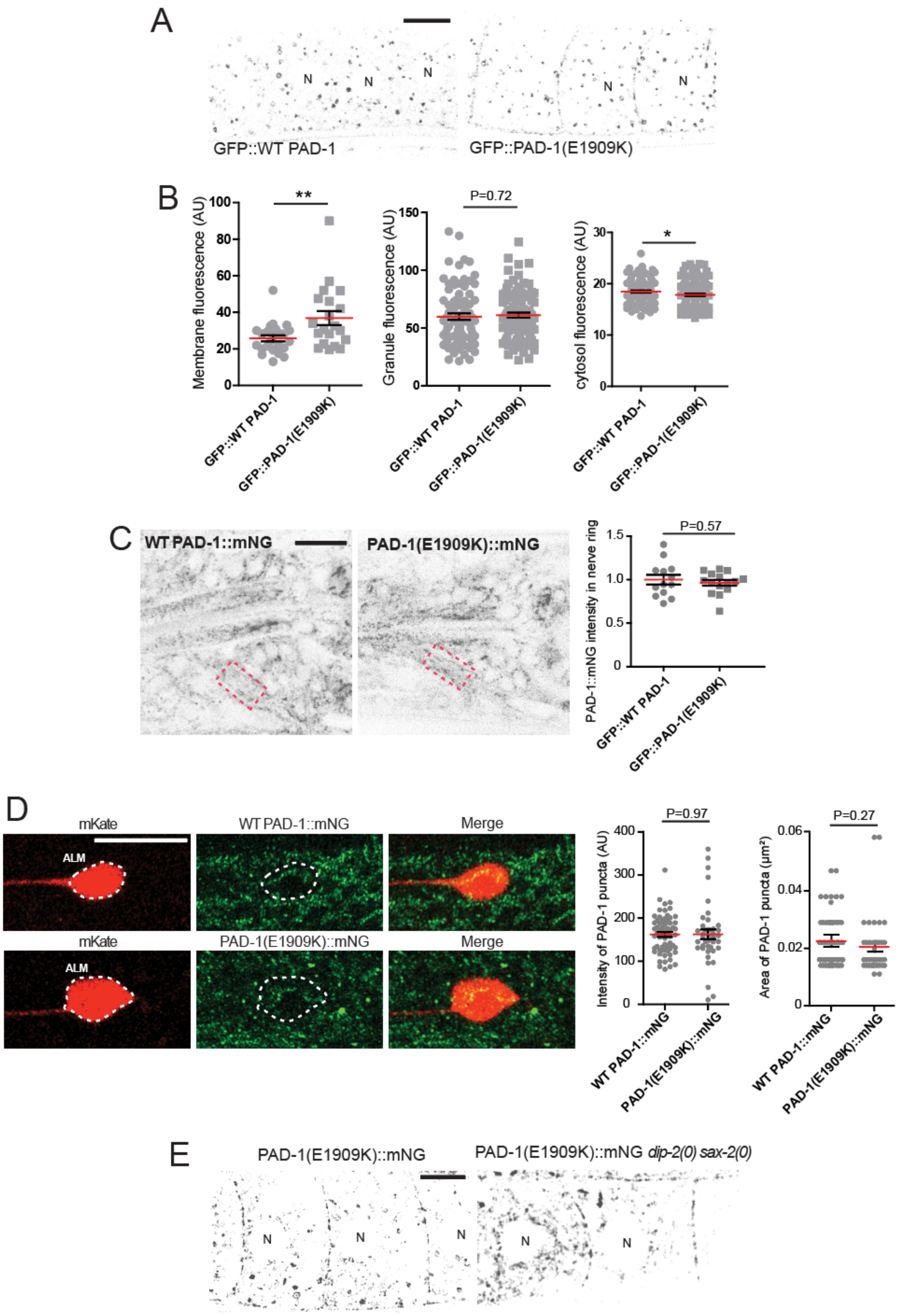

bioRxiv preprint doi: https://doi.org/10.1101/2024.05.07.591898; this version posted May 8, 2024. The copyright holder for this preprint (which was not certified by peer review) is the author/funder, who has granted bioRxiv a license to display the preprint in perpetuity. It is made available under aCC-BY 4.0 International license.

Supplemental Figure 6. E1909K gain-of-function mutation augments PAD-1's association with the plasma membrane in oocytes and the nervous system
(A,B) Confocal images and quantitation of GFP::PAD-1 localization and intensity. GFP::PAD-1 fluorescence at oocyte plasma membranes was significantly increased by E1909K; localization to vesicles/granules was unaffected and localization to cytosol was decreased. Dot plots show mean (red bar) and SEM (black). Statistics: t-test. ** (P<0.01),* (P<0.05). (C) Confocal images (lateral views) of anterior ganglia and nerve ring (red dashed boxes) and quantitation of normalized PAD-1::mNG expression levels in the boxed regions. E1909K did not increase PAD-1 nerve ring localization (t test). (D) Confocal images of ALM neurons labelled with mKate and PAD-1::mNG KI (WT and E1909K). Quantitation of PAD-1::mNG puncta intensity (arbitrary units, AU) and puncta size in ALM soma. Localization of PAD-1::mNG in ALM was not altered by E1909K. (E) Confocal images of PAD-1(E1909K)::mNG in oocytes where PAD-1 puncta were found in the cytoplasm and plasma membrane. The patchy pattern of PAD-1(E1909K)::mNG puncta in oocyte plasma membrane was not altered in *dip-2(0) sax-2(0)* mutant background. For panels (A,C,D, and E), scale = 10 μm.

bioRxiv preprint doi: https://doi.org/10.1101/2024.05.07.591898; this version posted May 8, 2024. The copyright holder for this preprint (which was not certified by peer review) is the author/funder, who has granted bioRxiv a license to display the preprint in perpetuity. It is made available under aCC-BY 4.0 International license.

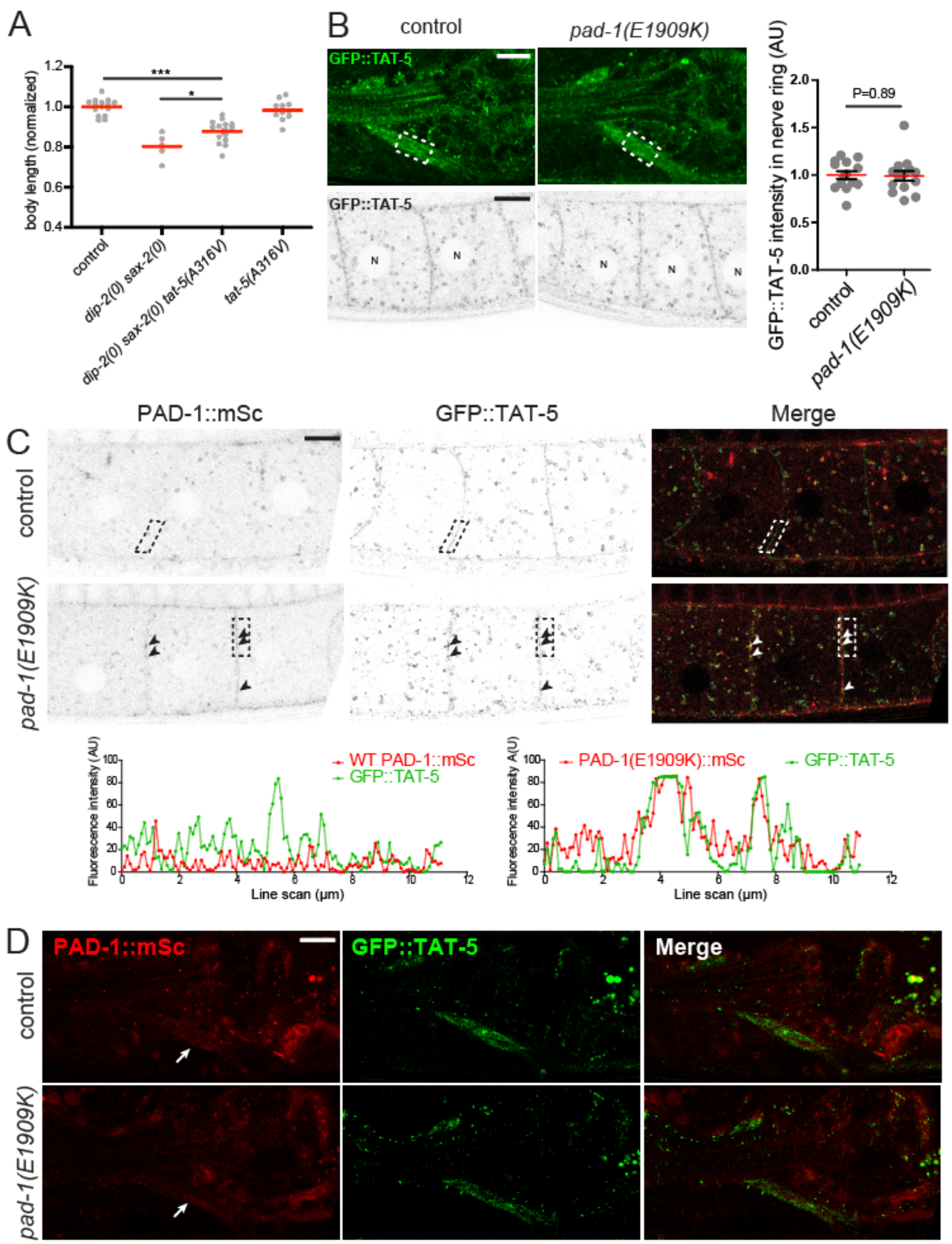

Supplemental Figure 7. Suppression of *dip-2 sax-2* phenotypes by *tat-5(A316V)* and TAT-5 localization in *pad-1(E1909K)*

(A) Quantitation of normalized body length measured in 1-day old adults. *tat-5(ju1878 A316V)* partially suppressed the reduced body length of *dip-2(0) sax-2(0)* double mutants and had normal body length as single mutant. Statistics: one-way ANOVA with Tukey's post test. *** (P<0.001),* (P<0.05). (B) Confocal images of GFP::TAT-5B/D KI (*wur36*) in the nervous system (*top*) and oocytes (*bottom*) and quantitation of

bioRxiv preprint doi: https://doi.org/10.1101/2024.05.07.591898; this version posted May 8, 2024. The copyright holder for this preprint (which was not certified by peer review) is the author/funder, who has granted bioRxiv a license to display the preprint in perpetuity. It is made available under aCC-BY 4.0 International license.

GFP::TAT-5B/D in nerve ring (*right*). GFP::TAT-5 localized to axons in the nerve ring (dashed boxes). Dot plot, normalized fluorescence signals of GFP::TAT-5 in the nerve ring. GFP::TAT-5 nerve ring localization was normal in *pad-1(E1909K)* mutants (t test). In oocytes, GFP::TAT-5 localized to punctate vesicles and close to the plasma membrane; this localization was similar in *pad-1(E1909K).* Oocyte nuclei indicated by N. (C) PAD-1(E1909K)::mSc displayed increased co-localization with GFP::TAT-5 at the plasma membrane (confocal images; black arrow heads; white arrow heads in merge); both PAD-1(E1909K)::mSc and GFP::TAT-5 displayed patchy localization at the oocyte plasma membrane. Line scans of fluorescence at the plasma membrane within dashed boxes were plotted using Fiji to show increased co-localization of PAD-1(E1909K)::mSc with GFP::TAT-5. (D) GFP::TAT-5 localization in the nerve ring area was normal in PAD-1(E1909K)::mSc animals. White arrows indicate nerve ring. For all panels, scale = 10 µm.
